# A large infrapatellar fat pad protects against knee pain and lateral tibial cartilage volume loss

**DOI:** 10.1186/s13075-015-0831-y

**Published:** 2015-11-10

**Authors:** Andrew J. Teichtahl, Ema Wulidasari, Sharmayne R. E. Brady, Yuanyuan Wang, Anita E. Wluka, Changhai Ding, Graham G. Giles, Flavia M. Cicuttini

**Affiliations:** Department of Epidemiology and Preventive Medicine, School of Public Health and Preventive Medicine, Monash University, Alfred Hospital, Melbourne, VIC 3004 Australia; Baker IDI Heart and Diabetes Institute, Commercial Road, Melbourne, VIC 3004 Australia; Menzies Research Institute Tasmania, University of Tasmania, Hobart, TAS 7000 Australia; Centre for Epidemiology and Biostatistics, Melbourne School of Population and Global Health, The University of Melbourne, Carlton, VIC 3053 Australia; Cancer Epidemiology Centre, Cancer Council Victoria, Melbourne, VIC 3004 Australia

**Keywords:** Infrapatellar fat pad, Knee, Osteoarthritis, Cartilage, Pain

## Abstract

**Introduction:**

The infrapatellar fat pad (IPFP) is commonly resected during knee joint arthroplasty, but the ramifications of doing so are unclear. This longitudinal study determined whether the size of the IPFP (maximum cross-sectional area (CSA)) was associated with knee cartilage loss and the development of knee pain in adults without knee osteoarthritis (OA).

**Methods:**

A total of 297 adults without American College of Rheumatology clinical criteria for a diagnosis of knee OA were recruited. Knee MRI was performed at baseline and an average of 2.3 years later. IPFP maximal CSA and tibial cartilage volume were measured from MRI. A large and small IPFP were defined by the median split, with a large IPFP defined by being in the highest 50 %. Body composition was performed at baseline using bio-impedance. Knee pain was assessed at follow-up using the Western Ontario and McMaster University Osteoarthritis Index (WOMAC).

**Results:**

A larger IPFP at baseline was associated with reduced knee pain at follow-up (OR 0.5, 95 % CI: 0.3 to 0.9, *p* = 0.02) and lateral tibial cartilage volume loss (β: −0.9 % (95 % CI: −1.6, −0.1 %) per annum, *p* = 0.03). The maximal CSA of the IPFP was predominantly located in the lateral (54.2 %), rather than the medial tibiofemoral compartment (1.7 %). Male gender (OR 12.0, 95 % CI: 6.5 to 22.0, *p* < 0.001) and fat free mass (OR 1.15, 95 % CI 1.04 to 1.28, *p* = 0.007) were both associated with a large IPFP.

**Conclusion:**

A larger IPFP predicts reduced lateral tibial cartilage volume loss and development of knee pain and mechanistically might function as a local shock-absorber. The lack of association between measures of adiposity and the size of the IPFP might suggest that the IPFP size is not simply a marker of systemic obesity.

## Introduction

Knee osteoarthritis (OA) is a whole organ disease, with abnormalities in bone, cartilage, menisci, ligaments and muscular supports [1]. An underappreciated structure in epidemiological studies of knee OA is the infrapatellar fat pad (IPFP). The IPFP, also known as the Hoffa fat pad, is an intracapsular but extrasynovial structure [2] that akin to subcutaneous adipose tissue, is capable of secreting multiple pro-inflammatory cytokines [3]. As well as having a pro-inflammatory role that may be important in the pathogenesis of knee OA, the close proximity of the IPFP to articular cartilage and bone may enable it to function as a shock-absorber, cushioning the impact of joint loading through the knee.

Despite the clinical consequences of IPFP excision being poorly understood, the IPFP is often surgically removed to improve the view of the tibial plateau when performing total knee arthroplasty [4]. Nevertheless, the IPFP is innervated by nociceptive nerve fibers, and may be an important structural determinant of knee pain [3]. In a retrospective observational study of people who predominantly had knee OA, IPFP resection resulted in increased knee pain [5]. Similarly, in a randomized controlled trial of IPFP resection or preservation at total knee arthroplasty for rheumatoid arthritis, increased anterior knee pain was noted after IPFP resection [6]. These data support the concept that the preservation of the IPFP may help to attenuate knee pain.

As well as symptoms, preservation of the IPFP may help to maintain the integrity of the knee structures. In one recent cross-sectional study, an increased maximal cross-sectional area (CSA) of the IPFP was associated with reduced radiographic joint space narrowing, osteophytes, increased cartilage volume, and reduced risk of cartilage defects and bone marrow lesions [7]. In the only longitudinal study, a larger CSA of the IPFP was associated with reduced tibial cartilage volume loss and reduced risk of medial cartilage defects over 2.6 years in females only [8]. This has yet to be corroborated in a separate population. Furthermore, the potential mechanism for this effect is unclear. In cross-sectional analyses, it was hypothesised that the beneficial associations between a larger IPFP and knee pain and structure might be due to enhanced shock-absorption capabilities imparted by a larger IPFP [7]. However, as the IPFP has been compared to subcutaneous adipose tissue capable of secreting pro-inflammatory cytokines [3], any increase in fat mass would likely be reflected by a larger IPFP and detrimental change in the cartilage. Examining the relationship between the IPFP and body composition may therefore help us to better understand the function of the IPFP.

The aim of this prospective cohort study was to examine the associations between the maximal CSA of the IPFP and changes in knee cartilage volume and knee pain in community-based adults without diagnosed knee OA. We also aimed to examine the relationships between the IPFP and obesity and body composition.

## Methods

### Subjects

The study was conducted within the Melbourne Collaborative Cohort Study (MCCS) as previously described [9]. We invited subjects who attended the first year of the second follow up of the MCCS, which commenced in 2003 provided they were aged between 50 and 79 years and did not meet a clinical diagnosis of knee OA as defined by American College of Rheumatology criteria [10]. Participants were also excluded if they indicated a history of knee pain lasting for >24 h in the last 5 years; a previous knee injury requiring non-weight bearing treatment for >24 h or surgery (including arthroscopy); or a history of any arthritis diagnosed by a medical practitioner. A further exclusion criterion was any contraindication to magnetic resonance imaging (MRI). We used quota sampling whereby we stopped recruitment when we reached our target sample of approximately 300 subjects. Follow-up MRI was performed between 2006 and 2007, with an average of 2.3 years having elapsed since the initial imaging study. The study was approved by the Human Research Ethics Committees of The Cancer Council Victoria and Monash University. All participants gave written informed consent.

### Anthropometric measures

Height (cm) was measured at baseline using a stadiometer, whereby the subject was asked to remove any footwear. Body mass (kg) was measured on entry to the current study, with bulky clothing removed. Body mass index (BMI) was calculated from these data (body mass (kg)/height^2^ (m^2^)).

### MRI

In 2003–2004 and again in 2006–2007, each subject had MRI performed on their dominant knee (defined as the lower limb the subject used to kick a ball). Knees were imaged in the sagittal plane on a 1.5-T whole body magnetic resonance unit (Philips 1.5 Tesla Intera; Philips Medical Systems, Eindhoven, The Netherlands) using a commercial transmit-receive extremity coil. The following sequence and parameters were used: a T1-weighted fat-suppressed 3D gradient recall acquisition in the steady state; flip angle 55°; repetition time 58 msec; echo time 12 msec; field of view 16 cm; 60 partitions; 512 × 512 matrix; one acquisition time 11 min 56 sec. Sagittal images were obtained at a partition thickness of 1.5 mm and an in-plate resolution of 0.31 × 0.31 mm (512 × 512 pixels).

**Fig. 1:**
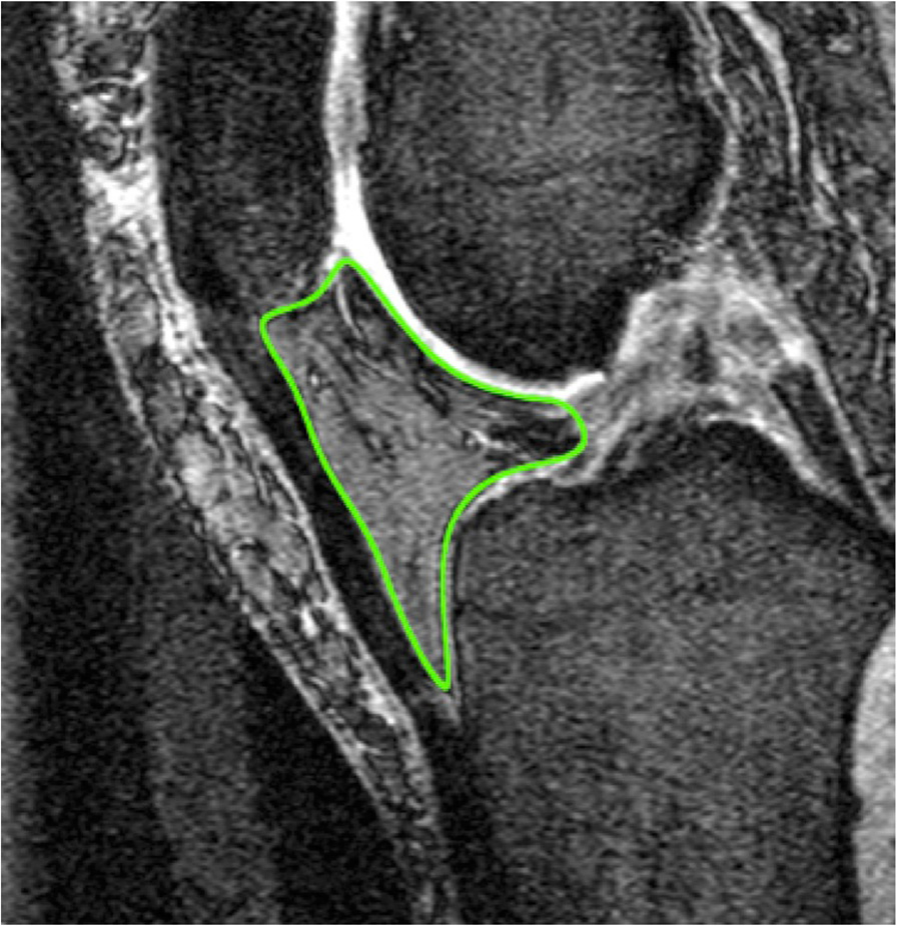
Sagittal representation of the maximal cross-sectional area (CSA) of the infrapatellar fat pad of the (IPFP) (outlined)

IPFP was measured by manually drawing disarticulation contours around the IPFP boundaries on section-by-section T1-weighted sagittal MR images, using the software program Osiris (University of Geneva). Computed single slices were reviewed to find the maximal CSA. The maximal CSA (cm^2^) was selected to represent the IPFP size (Fig. 1). One observer measured the IPFP area on all MR images with random cross checks performed by a second independent observer. The intra-class correlation coefficient (ICC) was 0.96 for intra-observer reliability (measured in 40 images by one observer), and inter-observer reliability was 0.92 (measured in 40 images by two observers) [7, 8]. The maximal CSA measured in the sagittal plane was then transposed on the axial plane. From the axial image, it was determined whether the maximal CSA was aligned with 1) the medial tibiofemoral, 2) the lateral tibiofemoral or 3) the midline (neutral). The maximal CSA of the IPFP was assessed for normality, and it was not normally distributed (*p* <0.0001 for Shapiro-Wilk test). Therefore, the median value was used to determine small and large maximal CSA of the IPFP at baseline.

Cartilage volume and bone area (volume at the patella) were determined by image processing on an independent workstation using the Osiris software. Contours were drawn around the patella on images 1.5 mm apart on sagittal views. Patellar cartilage volume was isolated from the total volume by manually drawing a disarticulation contour around the cartilage boundaries on each section. Patellar bone volume was determined by drawing contours around the patella boundaries in images 1.5 mm apart on sagittal views in a similar fashion to that described for cartilage volume. The coefficients of variation were 2.1 % for patellar cartilage volume and 2.2 % for patellar bone volume [11]. The volumes of the individual cartilage plates (medial and lateral tibial) were measured from the total volume by manually drawing disarticulation contours around the cartilage boundaries on each section. The volume of the particular cartilage plate was determined by summing the pertinent voxels within the resultant binary volume. A trained observer read each MR image. Independent measures of volume were made in a blinded fashion by a second trained observer. The coefficients of variation (CVs) for the medial and lateral cartilage volume measures were 3.4 and 2.0 %, respectively. Medial and lateral CSAs of the tibial plateau were determined by creating an isotropic volume from the input images, which were reformatted in the axial plane. Areas were directly measured from these images. CVs for the medial and lateral tibial plateau areas were 2.3 and 2.4 %, respectively.

### Knee pain

At follow up, pain was assessed by the Western Ontario and McMaster University Osteoarthritis Index (WOMAC) [12], which is widely used in community-based studies of adults [12, 13]. The pain subscales comprise five questions (walking on a flat surface, going up/down stairs, at night while in bed, when sitting/lying, and when standing upright), each assessed on a 100-mm visual analog scale (VAS), and summed to give a total score out of 500. Increase in score corresponds with worsening of pain. As participants were excluded at baseline if they indicated a history of knee pain lasting for >24 h in the last 5 years or had a history of any arthritis diagnosed by a medical practitioner, knee pain was assessed at follow up. WOMAC pain at follow up was assessed for normality and found not to be normally distributed (*p* <0.0001 for Shapiro-Wilk test). Therefore, the median value was used to determine high and low WOMAC knee pain at follow up.

### Body composition

Bioelectric impedance analysis was performed at baseline with a single frequency (50 kHz) electric current produced by a BIA-101A RJL system analyzer (RJL systems, Detroit, MI, USA). Resistance and reactance were measured with subjects in the supine position. The non-adipose mass, hereafter termed fat-free mass (kg), was estimated as 9.1536 + (0.4273 × height^2^/resistance) + (0.1926 × weight) + (0.0667 × reactance) for male subjects, and as 7.7435 + (0.4542 × height^2^/resistance) + (0.119 × weight) + (0.0455 × reactance) for female subjects [14]. The adipose mass, hereafter termed fat mass (FM) (kg) (FM = weight – fat-free mass) was subsequently calculated.

### Statistical analyses

With a sample size of 271, we had a power of 80 % to detect correlation as low as 0.2 between the IPFP and annual percentage change in cartilage volume (alpha error 0.05, two-sided significance). The annual average change in volume calculated between 2003–2004 and 2006–2007 was determined by the following equation:

$$ \frac{Baseline- Follow\  up}{\frac{Baseline}{Time\; between\; scans}}\times 100 $$

Cartilage volumes and the average annual change in cartilage volumes between 2003–2004 and 2006–2007 were assessed for normality (i.e., by checking for conformity with a bell-shaped distribution and performing the Wilk-Shapiro test) prior to linear regression analyses where these variables were the outcome. Multiple linear regression models were constructed to describe the relationships between the maximal CSA of the IPFP at baseline and both baseline and annual percentage change in cartilage volumes, adjusting for the potential confounders of age, gender, BMI and respective tibial plateau bone area (bone volume at the patella). When either the IPFP or pain at follow up were the dependent outcomes, binary logistic regression was used. *P* values <0.05 were considered to be statistically significant. All analyses were performed using the SPSS statistical package (standard version 20.0, SPSS, Chicago, IL, USA).

## Results

Subject characteristics are shown in Table 1. The mean age of the subjects at baseline was 58.0 (±5.5) years, with the cohort being predominantly comprised of women (62.6 %). On average, participants were overweight, with a mean BMI of 25.9 (±4.3) kgm^−2^. There were no significant differences in the baseline characteristics of the people who did and did not complete follow up (data not shown). The axial location of the maximal CSA of the IPFP was predominantly in the lateral tibiofemoral compartment (n = 161; 54.2 % of the cohort) or neutral (n = 127 or 42.8 % of the cohort) and rarely in the medial tibiofemoral compartment (n = 5 or 1.7 % of the cohort). Whereas 64.9 % of male subjects (n = 72) had a laterally located maximal CSA of the IPFP, the proportion of female subjects with a laterally located maximal CSA of the IPFP (48.8 %, n = 91) was significantly smaller (*p* = 0.017).

**Table 1:** Subject characteristics

	Baseline (n = 297)	Follow up (n = 271)
Age	58.0 (5.5)	60.2 (5.2)
Gender, female, n (%)	186 (62.6)	169 (62.4)
Body mass index, kgm^−2^	25.9 (4.3)	25.8 (4.0)
WOMAC pain measures	-	
Mean		34.6 (41.1)
Low, range in mm		0.0–24.9
High, range in mm		25.0–247.00
IPFP measures		
IFPF maximal cross-sectional area, cm^2^	6.9 (1.2)	-
Small, range in cm^2^	4.22–6.72	
Large, range in cm^2^	6.73–10.57	
Cartilage volume, mm^3^		
Medial tibial	1,705 (530)	1,651 (507)
Lateral tibial	2,026 (644)	1,991 (626)
Patella	2,918 (941)	2,807 (937)
Tibial bone area, mm^2^		
Medial	2,014 (309)	-
Lateral	1,288 (195)	-
Patella bone volume, mm^3^	20,276 (4,667)	-
Change data		
Annual percentage cartilage loss (SEM)		
Medial tibial	1.2 (0.2)	
Lateral tibial	1.1 (0.2)	
Patella	1.8 (0.2)	

Results displayed as mean (standard deviation) unless otherwise stated. *WOMAC* Western Ontario and McMaster University Osteoarthritis Index, *IPFP* infrapatellar fat pad, *SEM* standard error of the mean

The associations between a large IPFP at baseline and cartilage volumes are shown in Table 2. Cross-sectional analyses at baseline demonstrated that a larger IPFP CSA tended to be associated with a greater volume of medial (*β* 81 mm^3^, 95 % CI −11 to 173 mm^3^, *p* = 0.08) and lateral (*β* 90 mm^3^, 95 % CI −22 to 202 mm^3^, *p* = 0.11) tibial cartilage after adjustment for age, gender, BMI and respective tibial bone area. A larger IPFP at baseline was associated with a reduced rate of lateral tibial cartilage volume loss (*β* −0.9 %, 95 % CI −0.6 to 00.1 %, *p* = 0.03) after adjusting for gender, baseline age, BMI and lateral tibial plateau bone area. There were no significant associations between a large IPFP and medial tibial or patellar cartilage volume loss. The associations between a larger baseline IPFP and knee pain are also shown in Table 2. Subgroup analyses were performed according to the axial location of the maximal CSA of the IPFP (data not shown). Where the axial location of the maximal CSA of the IPFP was in the lateral tibiofemoral compartment, a larger IPFP tended to be associated with a reduction in the rate of lateral tibial cartilage volume loss (*β* −1.0 %, 95 % CI −2.5 to −0.1 %, *p* = 0.04), but this relationship was non-significant when the axial location of the maximal CSA of the IPFP was neutrally located (*β* −0.5 %, 95 % CI −1.9 to 0.9 %, *p* = 0.48) after adjusting for age, gender, BMI and lateral tibial bone area. A larger IPFP at baseline was associated with a reduced risk of a high level of knee pain at follow up (odds ratio (OR) 0.5, 95 % CI 0.3 to 0.9, *p* = 0.02).

**Table 2:** Associations between a large baseline IPFP and cartilage volume and knee pain

	Univariate analysis	*P*	Multivariate analysis	*P*
Baseline IPFP, cm^2^ and cartilage volume, mm^2^ β (95 % CI)				
Medial tibial^a^	516 (408, 623)	<0.001	81 (−11, 173)	0.08
Lateral tibial^a^	608 (476, 739)	<0.001	90 (−22, 202)	0.11
Patella^b^	835 (657, 1014)	<0.001	−3 (−162, 156)	0.97
Annual % change in cartilage volume				
Medial tibial^a^	0.0 (−0.8, 0.7)	0.93	−0.1 (−1.0, 0.8)	0.79
Lateral tibial^a^	−0.5 (−1.1, 0.1)	0.13	−0.9 (−1.6, −0.1)	0.03
Patella^b^	0.0 (−0.7, 0.7)	0.94	0.5 (−0.4, 1.4)	0.27
Knee pain at follow up				
High pain^c^, odds ratio (95 % CI)	0.7 (0.4, 1.1)	0.09	0.5 (0.3, 0.9)	0.02

Median infrapatellar fat pad (*IPFP*) maximal cross-sectional area value used to determine large and small IPFP size. Median WOMAC pain value used to determine high and low knee pain assessed by Western Ontario and McMaster University Osteoarthritis Index at follow up. ^a^Multivariate adjusted for gender, baseline age, body mass index (BMI) and respective tibial plateau bone area (mm^2^). ^b^Multivariate adjusted for gender, baseline age, BMI and patellar bone volume (mm^3^). ^c^Multivariate adjusted for gender, baseline age and BMI

The associations between a large IPFP and age, gender, BMI and body composition measures are shown in Table 3. Compared to female subjects, male subjects had a significantly larger IPFP (OR 12.0, 95 % CI 6.5 to 22.0, *p* <0.001) after adjusting for age and BMI. Although BMI (OR 0.99, 95 % CI 0.93 to 1.06, *p* = 0.84) and fat mass (OR 0.97, 95 % CI 0.93 to 1.02, *p* = 0.22) were not associated with a large IPFP size, increased fat-free mass was associated with a larger IPFP (OR 1.15, 95 % CI 1.04 to 1.28, *p* = 0.007) after adjusting for age, gender and fat mass (kg).

**Table 3:** Associations between subject age, gender and body composition and the risk of a large infrapatellar fat pad (IPFP)

	Univariate odds ratio (95 % CI)	*P*	Multivariate odds ratio (95 % CI)	*P*
Maximal cross-sectional area, cm^2^				
Age^a^, years	1.01 (0.97, 1.05)	0.70	0.97 (0.92, 1.02)	0.25
Male^a^ gender	11.1 (6.2, 20.0)	<0.001	12.0 (6.5, 22.0)	<0.001
BMI^a,^ kg m^−2^	1.02 (0.97, 1.08)	0.44	0.99 (0.93, 1.06)	0.84
Fat mass^b^, kg	1.00 (0.97, 1.03)	0.81	0.97 (0.93, 1.02)	0.22
Fat-free mass^c,^ kg	1.16 (1.12, 1.21)	<0.001	1.15 (1.04, 1.28)	0.007

Median IPFP maximal cross-sectional area value used to determine large and small IPFP size. ^a^Multivariate adjusted for gender, age and body mass index (*BMI*). ^b^Multivariate adjusted for gender, age and fat free mass (kg). ^c^Multivariate adjusted for gender, age and fat mass (kg)

## Discussion

This longitudinal study has demonstrated that a larger IPFP is associated with reduced knee pain and lateral tibial cartilage volume loss. The predilection for the IPFP to prevent cartilage loss at the lateral tibia may relate to the maximal CSA predominantly residing within the lateral knee joint, providing a local shock-absorbing mechanism. The lack of association between measures of adiposity and the size of the IPFP might suggest that the IPFP size is not simply a marker of systemic obesity.

In this study, a larger baseline IPFP tended to be positively associated with baseline medial (*p* = 0.08) and lateral (*p* = 0.11) tibial cartilage volumes. This finding is consistent with a previous cross-sectional study [7], and might be the consequence of body size, whereby the dimensions of one structure are reflected in the size of other structures. Longitudinally, a recent study demonstrated that the maximal CSA of the IPFP was associated with a reduction in the annual percentage change of both the medial and lateral tibial cartilage volume in women [8]. The current study has demonstrated that independent of gender, a larger maximal CSA of the IPFP is associated with a reduced rate of lateral tibial cartilage volume loss. We extended previous work [8] by demonstrating that the maximal CSA of the IPFP is either laterally (54.2 %) or neutrally (42.8 %) located, but rarely medially positioned (1.7 %). Such evidence helps to substantiate that the IPFP might function as a local shock-absorber at the lateral tibial cartilage, protecting it from increased joint loads and accelerated cartilage loss. Unlike the only previous longitudinal study, which showed greater CSA of the IPFP was associated with reduced medial cartilage volume loss in female subjects only [8], we did not show that a larger IPFP protects against medial tibial cartilage loss. In this study, post-hoc analyses demonstrated that female subjects were less likely than male subjects to have a laterally located maximal CSA of the IPFP (48.6 % versus 65.5 %, *p* = 0.017). The greater medial-lateral variability in the location of the IPFP among female subjects may be one reason that female subjects with a larger IPFP may be protected both from accelerated medial and accelerated lateral tibial cartilage volume loss.

As well as protecting lateral cartilage volume from accelerated loss, a larger IPFP was associated with reduced knee pain at follow up. A recent small (n = 46) cross-sectional study examining people with patellofemoral OA found that a larger IPFP volume was associated with greater knee pain [15]. However, the cross-sectional design limits any inferences to the IPFP being a cause of knee pain. Indeed, in longitudinal work, a larger IPFP CSA was associated with an improvement in knee pain in women [8]. It has been hypothesized that the IPFP might be important in pain generation, because nerve branches from numerous peripheral nerves traverse the structure [16], and contain substance P fibers [3, 17]. Signal changes in the IPFP are associated with fluctuations in knee pain among people with knee OA [18]. Although excision of the IPFP is common at knee arthroplasty [4], the consequences of this are unknown. In a retrospective observational study of people who predominantly had knee OA, IPFP resection resulted in increased knee pain [5]. In a randomized controlled trial of IPFP resection versus preservation at total knee arthroplasty for rheumatoid arthritis, increased anterior knee pain was noted after IPFP resection [6]. These and our data suggest that maintaining the IPFP may help to benefit knee structure and symptoms. The decision to remove the IPFP at surgery should be treated with caution.

As with previous studies, we did not find a significant association between the BMI and the maximal CSA of the IPFP [7, 8]. However, the BMI cannot discriminate adipose from non-adipose tissue. To our knowledge, no previous study has examined the association between body composition and the size of the IPFP. In this study we found that fat-free mass but not fat mass was associated with a larger IPFP. The lack of association between measures of adiposity and the size of the IPFP might suggest that the IPFP size is not simply reflected by systemic obesity. Nevertheless, the association between increased fat-free mass and a larger IPFP suggests that the size of the IPFP may be potentially modifiable by factors such as exercise.

The potential mechanism accounting for a beneficial symptom and structural effect imparted by a large IPFP remains speculative. In previous cross-sectional analyses, it was hypothesised that a larger IPFP may act as a shock-absorber to dissipate knee joint loads [8]. Our data support this, adding that the lateral tibial cartilage may be particularly protected secondary to the maximal CSA of the IPFP predominantly residing within this compartment. Indeed, in subgroup analyses, the association between the IPFP size and reduced lateral tibial cartilage volume loss was only apparent when the axial location of the maximal CSA of the IPFP resided within the lateral tibiofemoral compartment. Nevertheless, it must be acknowledged that the IPFP might exert its chondroprotective effect by systemic mediators. Like other adipose tissue, the IPFP has been postulated to have a metabolic and pro-inflammatory role [3], contributing to cytokine production within the knee [19], and may be responsible for higher levels of inflammatory cytokines and adipokines than subcutaneous fat [20]. Nevertheless, as an increased maximal CSA of the IPFP was associated with beneficial symptom and structural outcomes, and was not associated with fat mass, a pro-inflammatory mechanism imparted by systemic adiposity seems less likely from these data. Moreover, if the association between the IPFP and cartilage volume were driven by a systemic mechanism, then significant results would be expected in all knee compartments and not just restricted to the lateral knee joint.

This study has several limitations. Based on previous work [8], we chose to measure the maximal CSA of the IPFP, rather than volume. These two measures are strongly correlated, with one measure explaining 76 % of the variability in the other (*r*^2^ = 0.758, *p* <0.0001) when we examined this relationship using post-hoc analyses. Measuring the volume of a structure does not account for geographic variations in size within that structure, a factor that may be important given that our data infers that the benefit of a large IPFP may be via a local shock-absorbing effect. We have shown that the maximal CSA of the IPFP is predominantly located in the lateral knee compartment. Volumetric assessment of the IPFP does not capture these data. We have also performed analyses where we categorized the maximal CSA of the IPFP as large and small, using a median cut off. While this may have resulted in some non-differential misclassification, this is likely to have reduced our ability to demonstrate any significant associations. Finally, we have defined knee pain at follow up by the median cut off (25 mm out of 500 mm). This cut off was used to define significant pain, and although smaller changes (>0 mm) have been used in other studies examining the IPFP [8], smaller changes may be harder to reconcile as clinically significant. We did not collect pain data at baseline as recruitment for this study required participants to have no clinical diagnosis of knee OA or knee pain lasting longer than 24 h in the past 5 years. Therefore, any detection of knee pain at follow up may represent incident knee pain, and any misclassification from baseline would have only served to reduce the ability to show significant associations between baseline IFPF size and knee pain in this study.

## Conclusions

This longitudinal study has demonstrated that a larger IPFP is associated with reduced knee pain and lateral tibial cartilage volume loss. The predilection for the IPFP to prevent cartilage loss at the lateral tibia may relate to the maximal CSA predominantly residing within the lateral knee joint, providing a local shock-absorbing mechanism. The lack of association between measures of adiposity and the size of the IPFP might suggest that the IPFP size is not simply a marker of obesity, but may be modified by fat-free mass. The decision to remove the IPFP at surgery should be treated with caution, as it may be important in helping to prevent knee OA, particularly in the lateral compartment.

### Significance and innovation

A large IPFP prevents knee cartilage loss, mainly in the lateral compartment. It also helps to prevent knee pain. This protective affect may primarily be via cushioning. The lack of association between measures of adiposity and the size of the IPFP might suggest that the IPFP size is not simply a marker of systemic obesity. Caution when removing the IPFP at surgery is recommended, as it may be to the long-term detriment of the knee.

## Abbreviations

BMI: body mass index
CSA: cross-sectional area
CV: coefficient of variation
ICC: intra-class correlation coefficient
IPFP: infrapatellar fat pad
MCCS: Melbourne Collaborative Cohort Study
MRI: magnetic resonance imaging
OA: osteoarthritis
OR: odds ratio
VAS: visual analog Scale
WOMAC: Western Ontario and McMaster University Osteoarthritis Index
